# Acoustic monitoring yields informative bat population density estimates

**DOI:** 10.1002/ece3.11051

**Published:** 2024-02-22

**Authors:** Meredith L. Hoggatt, Clarissa A. Starbuck, Joy M. O'Keefe

**Affiliations:** ^1^ Department of Natural Resources and Environmental Sciences University of Illinois at Urbana‐Champaign Urbana Illinois USA

**Keywords:** acoustics, bats, monitoring, population density, random encounter model

## Abstract

Bat population estimates are typically made during winter, although this is only feasible for bats that aggregate in hibernacula. While it is essential to measure summer bat population sizes for management, we lack a reliable method. Acoustic surveys should be less expensive and more efficient than capture surveys, and acoustic activity data are already used as indices of population size. Although we currently cannot differentiate individual bats by their calls, we can enter call counts, information on signal and detection angles, and weather data into generalized random encounter models to estimate bat density. We assessed the utility of generalized random encounter models for estimating Indiana bat (*Myotis sodalis*) population density with acoustic data collected at 51 total sites in six conservation areas in northeast Missouri, 2019–2021. We tested the effects of year, volancy period, conservation area, and their interactions on estimated density. Volancy period was the best predictor, with average predicted density increasing 60% from pre‐volancy (46 bats/km^2^) to post‐volancy (74 bats/km^2^); however, the magnitude of the effect differed by conservation area. We showed that passive acoustic surveys yield informative density estimates that are responsive to temporal changes in bat population size, which suggests this method may be useful for long‐term monitoring. However, we need more information to choose the most appropriate values for the density estimation formula. Future work to refine this approach should include assessments of bat behavior and detection parameters and testing the method's efficacy in areas where population sizes are known.

## Graphical abstract

It is essential to measure summer bat population sizes for management, but we lack a reliable method. We assessed the utility of generalized random encounter models for estimating Indiana bat (*Myotis sodalis*) population density with acoustic data collected at 51 total sites in six conservation areas in northeast Missouri, 2019–2021. Acoustic surveys yield informative density estimates that are responsive to temporal changes in bat population size, which suggests this method may be useful for long‐term monitoring.

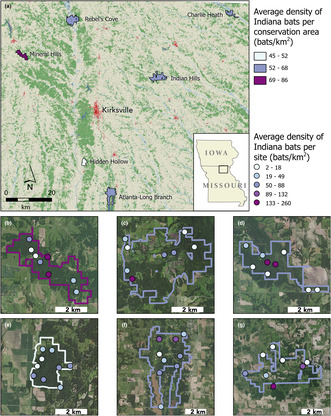

## 1 INTRODUCTION

Historically, bat population abundance has been estimated during the winter, because at this time, many species of bats aggregate and are relatively stationary. For example, Thogmartin et al. (2012) used winter count data to assess hibernacula‐level population trends for cave‐hibernating Indiana bats (*Myotis sodalis*) and to evaluate larger‐scale population declines due to white‐nose syndrome (WNS). Such an analysis was possible due to the contributions from agency biologists who conducted winter hibernacula counts in 24 states across the species' range from 1983 to 2009. While winter is the easiest time to count cave‐hibernating bats, this method misses bats that migrate to other regions, or hibernate cryptically—either hidden from view in large hibernacula or instead using small hibernacula that are not typically surveyed. Thus, in temperate regions, it would be beneficial to develop a method for measuring bat populations during the non‐hibernating period, when all bat species in an area are active.

Most temperate‐zone bats rely on forests during the non‐hibernation portion of their life cycle (Law et al., 2016), but forest‐dwelling bats exhibit behaviors that make it difficult to assess population sizes. All forest‐dwelling bats in the temperate region are cryptic, volant, and nocturnal. Most do not aggregate in large groups in natural roosts. For example, in North America, there are typically only about 25 individuals in a colony of crevice‐dwelling bats during summer (Lacki, 2018), and foliage‐roosting bats tend to roost in group sizes of <10 bats (Carter & Menzel, 2007). Furthermore, many species, including the endangered Indiana bat, have a fission‐fusion social structure in which colony members switch among multiple summer maternity roosts, making it difficult to locate individual bats or the colony (Callahan et al., 1997; Dambly et al., 2021; Humphrey et al., 1977; Silvis et al., 2014).

Established methods of assessing summer bat populations are labor‐intensive and costly, and all have biases. Methods for surveying bat populations include capturing bats (e.g., O'Keefe et al., 2019), conducting acoustic surveys (e.g., Barlow et al., 2015; Nocera et al., 2019a), and counting bats at known roost sites (e.g., Warren & Witter, 2002). Capture surveys are costly because they require trained surveyors, expensive equipment, and numerous hours of labor (Coleman et al., 2014). Also, some species successfully evade mist‐nets and are captured less frequently (Kunz & Brock, 1975; Larsen et al., 2007; Marques, Ramos Pereira, et al., 2013). Counting bats at known roosts generally first requires capture and the use of radio telemetry to locate roosts (e.g., O'Keefe & Loeb, 2017). Because roosts are often ephemeral (Barclay & Kurta, 2007) and many species regularly switch roosts (Kunz & Lumsden, 2003), it is not always feasible to estimate population sizes by counting bats at roosts. Acoustic surveys are less costly than capture surveys and have a higher probability of detecting cryptic species as they move about the landscape. For example, acoustic surveys yield greater species counts than mist‐net surveys (Robbins et al., 2008), and detection rates are higher with acoustic surveys than with systematic roost searches for two species of forest‐dwelling bats, Rafinesque's big‐eared bats (*Corynorhinus rafinesquii*) and southeastern myotis (*Myotis austroriparius*; Comer et al., 2014). However, acoustic surveys have biases and deficiencies: detection rates vary by species (Neuweiler, 1989), weather and environment affect recorded calls (Goerlitz, 2018), and identification methods are imperfect (Nocera et al., 2019b).

Despite this, because of improving technology and the lower cost of equipment and labor, monitoring programs such as the North American Bat Monitoring Program (NABat; Loeb et al., 2015) and the Norfolk Bat Survey (Newson et al., 2015) recommend using acoustic detectors to survey bat populations. Acoustic surveys also allow researchers to keep a permanent record of the survey data, which can be reanalyzed as identification methods evolve. As acoustics are incorporated into population monitoring surveys, it will be necessary to implement models accounting for biases associated with sampling cryptic, volant animals with dynamic foraging ranges and no unique signature in their calls. While methods for bat species identification are advancing (e.g., Khalighifar et al., 2022), we still cannot identify an individual bat based on its echolocation call (Weller, 2007).

Density estimation using acoustic data is a relatively new field with much promise, especially when animals are readily detectable acoustically, because this method is less constrained by survey time and weather, and data can be analyzed with automated systems (Marques, Thomas, et al., 2013). For example, Dähne et al. (2013) estimated harbor porpoise density using echolocation click loggers to measure population‐level responses to disturbances caused by driving piles for offshore wind turbine construction. Two recent studies have used acoustics to estimate bat population sizes without the need to recognize individuals. Kloepper et al. (2016) used the acoustic amplitude (root mean square sound pressure) for echolocation calls of Mexican free‐tailed bats (*Tadarida brasiliensis*) emerging from a cave to estimate the colony size. The acoustic amplitude was highly correlated with the number of bats counted coming out of the cave using a camera placed at the same site as the microphone (Kloepper et al., 2016). More recently, Milchram et al. (2019) adapted a generalized random encounter model (gREM) developed by Rowcliffe et al. (2008) to estimate the population density of forest bats from data collected using stationary acoustic detectors. Milchram et al. (2019) compared their estimates to published estimates for bat colony sizes and to the output from an evaluation of the same acoustic data with Royle–Nichols' detection/non‐detection models (Royle & Nichols, 2003). The generalized random encounter models and Royle–Nichols' models estimated lower densities than literature reports for two of three species, but the estimated density from the generalized random encounter models matched the published colony size density for *Pipistrellus* (Milchram et al., 2019).

Because of its endangered status and preference for ephemeral, dead trees as summer roost sites, the Indiana bat was a logical focal species for our study. Resource managers often want to know how management actions will affect populations of this sensitive species (e.g., Divoll et al., 2022), but Indiana bats are difficult to count during the summer because maternity colonies frequently divide into groups varying in size (e.g., Clement et al., 2015; O'Keefe & Loeb, 2017). Our research objectives were to (1) estimate the population size of Indiana bats in six conservation areas in northeastern Missouri and (2) validate the density estimates by assessing how factors such as time and space affect those estimates. We predicted that acoustic estimates of bat density would be higher than counts of individuals derived from concurrent capture and roost ecology work because bats sometimes avoid or escape mist nets (Robbins et al., 2008), and acoustic surveys are more likely to count the same individual multiple times (Fenton et al., 1973) and may be biased by false positives identifications, as discussed by Clement, Rodhouse, et al. (2014). Nonetheless, we predicted that if density estimates were at all related to actual population sizes, estimates would be higher during the second half of the summer due to the influx of volant pups and would vary among conservation areas due to differences in landscape size and habitat suitability.

## 2 MATERIALS AND METHODS

### 2.1 Study area

We conducted this study on six state‐owned conservation areas in northeast Missouri: Atlanta‐Long Branch Conservation Area, Charlie Heath Memorial Conservation Area, Hidden Hollow Conservation Area, Indian Hills Conservation Area, Mineral Hills Conservation Area, and Rebel's Cove Conservation Area. The study areas range in size from 550 to 1946 ha. Ongoing work has demonstrated that Indiana bats are roosting or foraging in all six conservation areas (Starbuck et al., 2022).

### 2.2 Survey design

We selected 10 acoustic sites per conservation area, for a total of 60 sites. We controlled for distance to water bodies to avoid recording in areas with inflated bat activity levels and to reduce the number of calls recorded for non‐target species that tend to forage over water. We allowed a minimum spacing among acoustic sites within the same conservation area to increase sampling coverage and reduce spatial autocorrelation. We used ArcMap version 10.7.1 (ESRI, Inc., Redland, CA, USA) to predetermine the general location of an acoustic site by generating random points within the conservation area and eliminating points that did not fit the following criteria: ≥50 m from non‐ephemeral waterbodies or flowlines; ≥100 m from flowlines that crossed the corridor being recorded, and ≥500 m away from another acoustic site. We delineated flowlines and waterbody areas in shapefiles provided by the National Hydrography Dataset (NHD; USGS, 2019); using Google Earth, we digitized any waterbodies or flowlines that were not included in the shapefiles but were visible through satellite imagery. We generated a list of points in random order, choosing acoustic sites on a forest edge facing a field or in an open corridor within a forest patch within 100 m of each generated random point, and skipping to the next point if we could not find a suitable site within this distance. We chose acoustic sites along forest edges (distance from detector position to opposite forest edge averaged 44.6 m, range 2.7–380 m) because Indiana bats mainly forage in or near forests (e.g., Divoll et al., 2022; Kniowski & Gehrt, 2014; Murray & Kurta, 2004), and edges serve as low‐clutter space for recording bat calls (Kaiser & O'Keefe, 2015).

At each site, we recorded bat echolocation calls with an Anabat Swift detector (version 1.6, Titley Electronics) coupled with a directional, stainless‐steel microphone housed in constructed polyvinyl chloride (PVC) weatherproofing. We set the microphone on a 3‐m high PVC pole, connected to a detector by a 10‐m cable, and at least 2 m from a tree to prevent foliage from affecting call quality. We tagged the tree for identification during future acoustic sampling periods. Along the horizontal axis, we directed the microphone at a 45° angle relative to the trail or field edge and, along the vertical axis, the microphone was oriented to be parallel to the ground. We randomly chose the direction of the microphone in relation to the field edge or corridor (i.e., left or right). We calibrated the microphones using a constant 40‐kHz tone; final sensitivity settings ranged from 13 to 15 to achieve equal detection rates for the tone. The detectors recorded full‐spectrum data in the preset nighttime mode, which records from 30 minutes before sunset until 30 minutes after sunrise. We chose the following settings for the detectors: constant recording = off; minimum frequency = 16 kHz; maximum frequency = 250 kHz; minimum event = 3 ms; trigger window = 2 s; sample rate = 500 kHz; max file length = 10 s; Analog HP filter = on; sdfilename prefix = off. Data were stored internally on two 32‐GB SanDisk SD cards and detectors were powered by four rechargeable AA batteries.

Each of the 60 survey sites was visited twice each summer (2019–2021), once during pre‐volancy (before bat pups are flying; May–June) and again during post‐volancy (when pups are flying; July–August; USFWS, 2007). During each survey period, we deployed the 10 detectors simultaneously in a conservation area with the goal of recording for at least four consecutive nights. However, due to equipment error, sometimes we did not meet this goal. For the analyses described herein, we retained the first four nights of any successful survey of ≥4 nights. We considered a site “complete” if we recorded for four nights during both pre‐ and post‐volancy periods in each of the 3 years. We had complete survey data for 8–9 sites per conservation area. For our analyses, we used bat acoustic data recorded at 51 sites for 1224 detector‐nights (1 unit active for 1 night).

We used a decision tree with a filter in Anabat Insight (Version 1.9.0‐5‐g38ddrr8; Titley Scientific, Columbia, MO, USA) to separate call files with Indiana bat echolocation characteristics (Clement, Murray et al., 2014; Murray et al., 2001). The filter had three parameters: characteristic frequency (the frequency at the flattest part of the pulse, Fc), characteristic slope (the slope at the flattest part of the pulse, Sc), and duration (the length of time a pulse is emitted, Dur). The filter settings were Fc = 38–55 kHz; Sc = 70–210 OPS; and Dur = 1–7 ms. We tested the filter accuracy on a set of known‐roost calls recorded by four Anabat Swift units across two nights during June 2020 within 5–10 meters of a known Indiana bat roost. Of 639 recorded known‐roost files, 565 were visually confirmed as Indiana bats; 461 of these passed the filter (81.6%). Known‐roost call files missed by the filter typically were social calls, muted calls with low bandwidth, or files containing multiple species, echoes, or other noise. The decision tree required that the call files passed the filter and had >5 pulses per call file to be classified as an Indiana bat. For all call files that passed the decision tree, we generated a report in Anabat Insight to get the count of 1‐minute time intervals per night that contained Indiana bat call files. We considered the number of 1‐minute intervals that contained ≥1 call files that passed the decision tree as the Indiana bat call count for a particular survey night. Because some bats may fly past and trigger the microphone repeatedly, the number of bat passes recorded may be a biased measure of acoustic activity; Miller (2001) demonstrated that species presence in a 1‐minute period is a more objective measure. We did not use a “manual vetting” process to review call files because of the high volume of data, and because automated identification methods are objective and repeatable (Marques, Thomas, et al., 2013).

### 2.3 Density estimate calculation

To estimate density based on call counts, we used the equations required for the generalized random encounter model detailed in Lucas et al. (2015); (Equations 1 and 2, Table 1). First, we needed to estimate the profile width (*p̄*), that is, the average width of the area in which a bat can be detected, a value that incorporates the width of the animal's signal, the detector's zone of reception, and the animal's angle of approach (Lucas et al., 2015). With Equation 1, we calculate *p̄*, where *r* is the detection distance (m), θ is the detection angle (°), and α is the acoustic signal angle (°) (Table 1). We estimated the detector threshold as −36 dB based on the expected typical detection distance for 40‐kHz calls of *Myotis* bats using the Anabat Swift detector and directional microphone (~30 m; C. Corben, pers. communication, 8 December 2021); our chosen threshold value was based on threshold values and detection distances for 40‐kHz bats as reported for EcoObs batcorder device used by Milchram et al. (2019) (ecoObs, 2023). To calculate detection distance (*r*) for Equation 1, we incorporated the detector threshold (dB), median nightly temperature (K), median nightly atmospheric pressure (kPa), median nightly relative humidity (%), main frequency of the bat call (Hz), and initial sound pressure level (dB) into the calculation for the atmospheric attenuation of sound (International Organization for Standardization, 1993; Milchram et al., 2019; Table 1). Equation 2 calculates density (*D*; bats/km^2^) over one recording night, where *z* is the number of detections, *v* is the average speed of the animal (m/s), and *t* is the recording time (s). We used our data and averaged values from the literature to fulfill parameters in the equations (Table 1). We calculated the density estimates in program R (R Core Team, 2020):

(1) $$\bar{p}=\frac{r}{\pi }\;\left(\theta \;\sin\frac{\alpha }{2}-\cos\frac{\alpha }{2}+1\right)$$

(2) $$D=\frac{z}{\mathrm{vt}\bar{p}}.$$

**TABLE 1:** The variables needed to calculate the density of Indiana bats (*Myotis sodalis*) per km^2^, the average width of the area in which a bat's call can be detected (*p̄*), and detection distance for a particular recording night (*r*) used in a study at six conservation areas in northeast Missouri, summers 2019–2021.

Equation	Parameter	Definition (units)	Value	Source
Density of bats at one site over one night (Lucas et al., 2015)				
*z*		Number of detections (number of recorded sequences between sunset and sunrise)	Varies	Own data; dependent on recording night
*v*		Average speed of animal (m/s)	3.9	Reference list below^a^
*t*		Recording time from sunset to sunrise (seconds)	Varies	Own data; dependent on recording night
*p̄*		Average width of the area in which a bat's call can be detected	Calculated	See below; dependent on recording night
Average width of the area in which a bat's call can be detected (*p̄*; Lucas et al., 2015)				
*r*		Detection distance (m)	Calculated	See below; dependent on recording night
*θ*		Detection angle (°)	20	C. Corben, Titley Scientific, (personal communication, 2021)
*α*		Acoustic signal angle (°)	31	Shimozawa et al., (1974); Surlykke et al., (2009); Jakobsen et al., (2013)
Detection distance for a particular recording night (*r*; Milchram et al., 2019)				
*T*		Median nightly temperature (K)	Varies	Kirksville airport weather station^b^
pa		Median nightly atmospheric pressure (kPa)	Varies	Kirksville airport weather station^b^
hr		Median nightly relative humidity (%)	Varies	Kirksville airport weather station^b^
*f*		Main frequency (Hz)	40,000	C. Corben, Titley Scientific, (personal communication, 2 November 2021)
Li		Initial sound pressure level (dB)	110	Milchram et al., (2019)
Threshold		Threshold value of detector (dB)	−36	C. Corben, Titley Scientific, (personal communication, 8 December 2021); M. Milchram, (personal communication, 5 November 2021)

^a^ Aizpurua et al. (2013); Aldridge (1988); Fawcett and Ratcliffe (2015); Grogzinski et al. (2009); Habersetzer and Vogler (1983); Hayward and Davis (1964); Jones and Rayner (1988); Kalko (1995); Kalko and Schnitzler (1989); Melcón et al. (2007); Miller and Treat (1993); Ratcliffe and Dawson (2003); Roby et al. (2019); Sunga et al. (2021); Surlykke et al. (1993); Womack et al. (2013).

^b^ National Weather Service data obtained from the Midwestern Regional Climate Center, cli‐MATE, Accessed 30 November 2023. https://mrcc.purdue.edu/CLIMATE/welcome.jsp.

### 2.4 Statistical analysis

We tested for the best‐fitting distribution for our data. The density estimates were skewed toward zero due to a high number of survey nights with zero counts, so we used a negative binomial distribution. To assess the validity of density estimates (response variable), we constructed six base generalized linear mixed effect models using three predictor variables—period, year, or conservation area—with site and date as random intercepts and the negative binomial family to account for overdispersion in the data. We also constructed a null model, a full additive model, and a model with period and conservation area as an interaction (Table 2). We used an information theoretic approach (Burnham & Anderson, 2002) to determine which models were plausible explanations (i.e., ∆AICc < 2) for the bat density estimates. All analyses were performed in Program R (R Core Team, 2020), and we used the *glmmTMB* package (Brooks et al., 2017) to fit mixed‐effects models, the *MuMIn* package (Bartón, 2009) to evaluate model fit, and the *lme4* package (Bates et al., 2015) to calculate confidence intervals.

**TABLE 2:** The generalized linear mixed effect models predicting the effects of volancy period, conservation area, and year on estimated Indiana bat density per km^2^ at six conservation areas in northeast Missouri, summers 2019–2021.

Model	K	ΔAICc	*w*_i_
Volancy period × conservation area	15	0.0	~1
Volancy period	5	21.9	<0.001
Volancy period + year + conservation area	12	29.1	<0.001
Null	4	56.2	<0.001
Year	6	59.8	<0.001
Conservation area	9	60.4	<0.001

*Note*: For each model, we show the number of parameters (K), the delta AICc value (ΔAICc), and the weight (*w*_*i*_).

### 2.5 Captures and roost ecology

In the summers of 2019 and 2021, we led a concurrent study that required capturing Indiana bats, tracking them to roosts using radio telemetry, and conducting exit counts at roost trees in each of the six conservation areas in pre‐ and post‐volancy periods (Starbuck et al., 2020, 2022). We compared our acoustic density estimates to the number of Indiana bats captured and maximum exit counts per conservation area across all periods in both 2019 and 2021.

## 3 RESULTS

**FIGURE 1:**
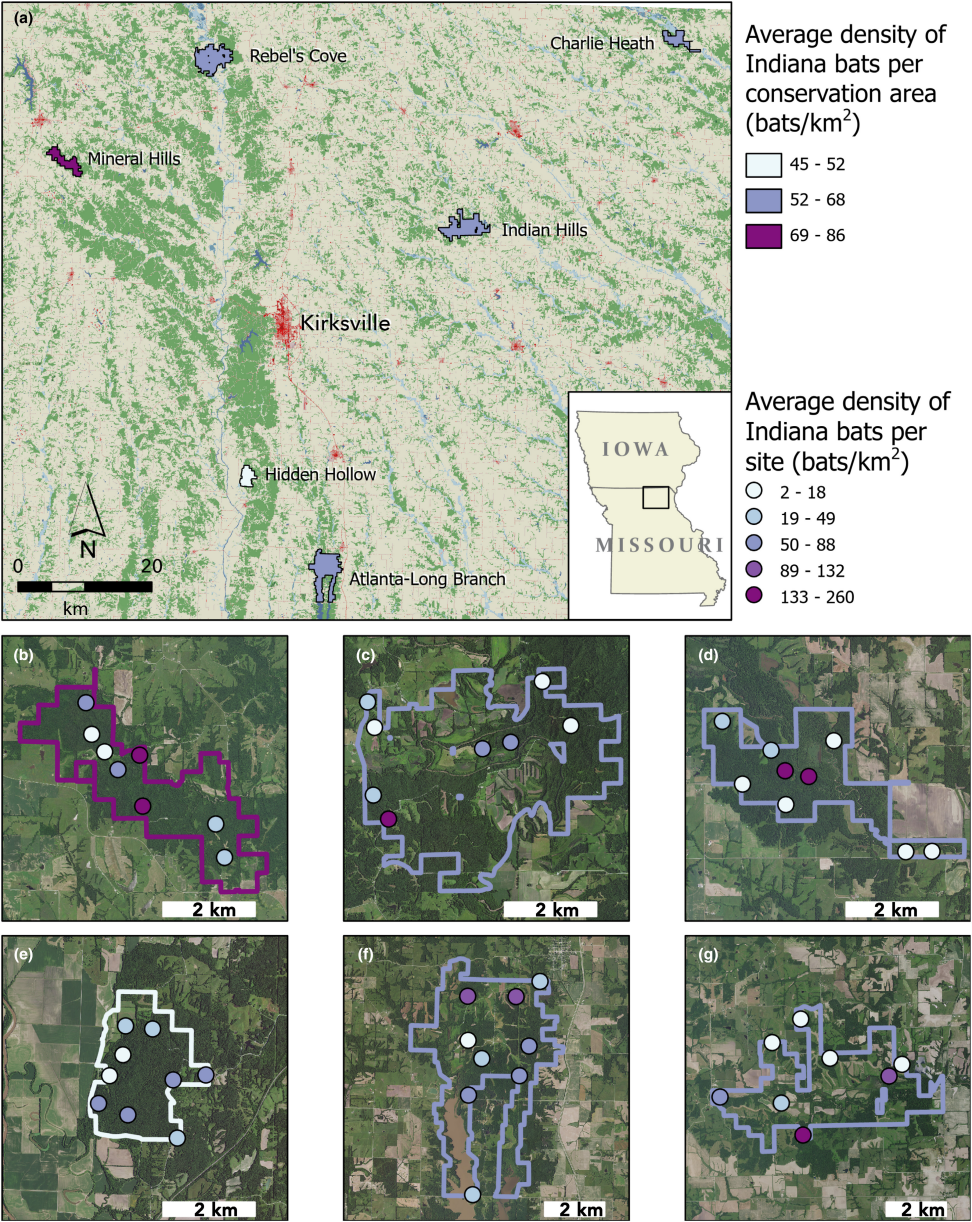
Six conservation areas in northeastern Missouri, USA in which we estimated Indiana bat (*Myotis sodalis*) density using acoustic surveys. Panel (a) shows the geographic position of the areas, which are centered on Kirksville, Missouri; polygon colors indicate average estimated Indiana bat density per conservation area over 3 years (2019–2021). The National Landcover Database‐derived basemap shows forested areas in green and developed areas in red hues (USGS 2023). Panels (b–g) show the average estimated Indiana bat density per site for each conservation area over 3 years (2019–2021); area outline shows overall area density, per Panel (a). Conservation area names by panel are: (b), Mineral Hills; (c), Rebel's Cove; (d), Charlie Heath Memorial; (e), Hidden Hollow; (f), Atlanta‐Long Branch; and (g), Indian Hills.

We recorded 312,059 call files in total; of these, 45,512 passed the Indiana bat filter. Density estimates ranged from 0 to 806 Indiana bats/km^2^ (per point per night, 51 points; Figure 1). Mean density estimates for each conservation area across all 3 years ranged from 45 to 85 bats/km^2^. Across all conservation areas and years, the mean density estimate during the pre‐volancy recording periods was 46 bats/km^2^, while during the post‐volancy recording periods it was 74 bats/km^2^.

**FIGURE 2:**
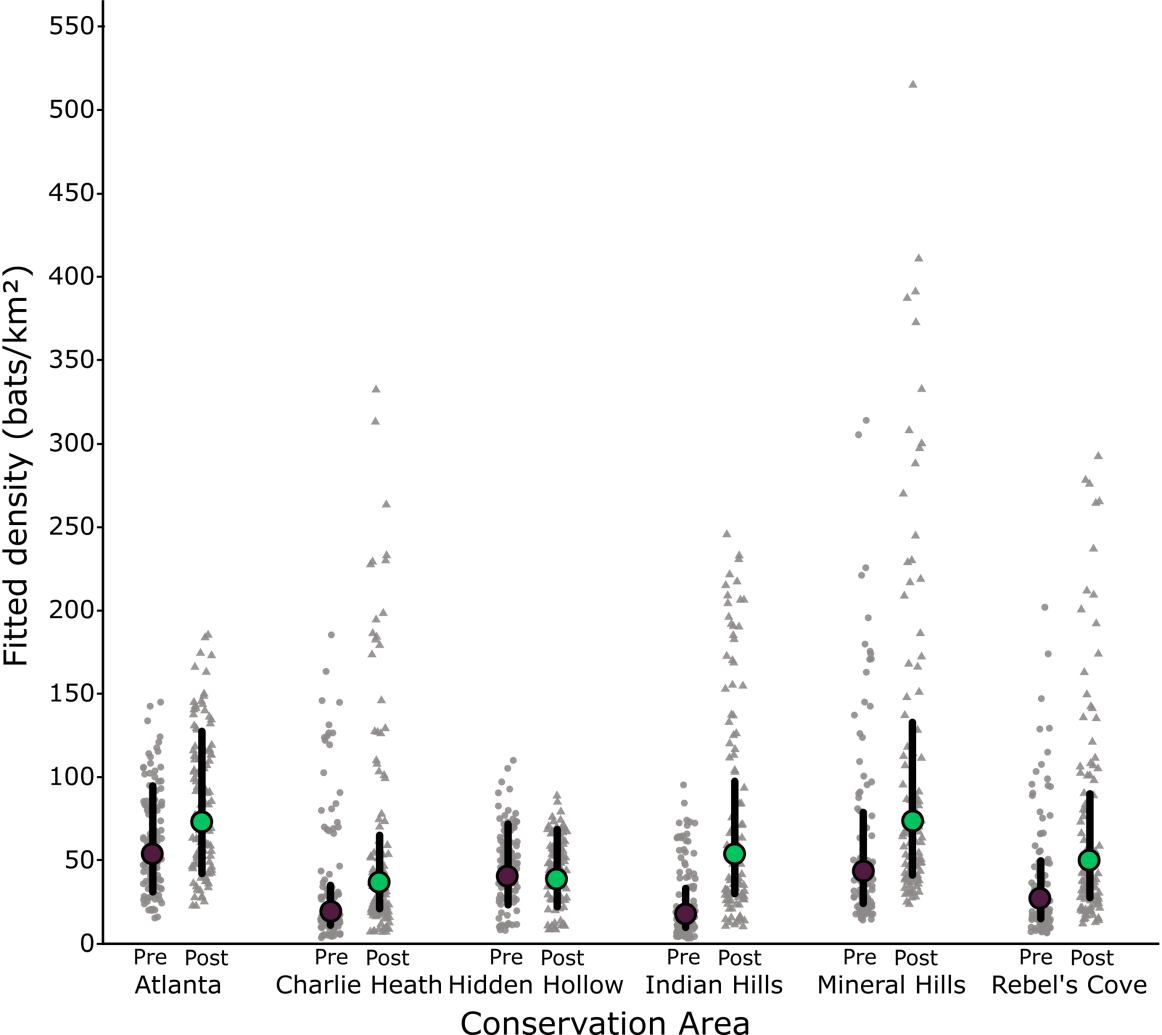
Fitted Indiana bat density estimates (bats/km^2^) for six conservation areas over 3 years (2019–2021) in northeastern Missouri, USA using data from acoustic surveys. Fitted density is shown for each of the two summer periods (pre‐volancy [before pups become volant] in purple with circle datapoints, and post‐volancy [after pups become volant] in green with triangle datapoints) for each surveyed conservation area. Each datapoint is the density estimate for one night of recording for a site. The colored dots represent the group mean across years and sites, and the lines represent 95% confidence intervals.

While we observed variation in density estimates among conservation areas and years, volancy period was the best predictor of density. Mean density estimates increased by 60% from pre‐volancy to post‐volancy (Figure 2). Only the volancy period × conservation area model was supported (Table 2), and the magnitude of the effect of volancy period differed depending on the conservation area (Figure 2).

High bat density estimates for particular conservation areas were driven by high‐density estimates at only one to two acoustic sites (Figure 1). For example, at Mineral Hills Conservation Area, average estimated bat densities were 261 (±30) and 182 (±36) bats/km^2^ for the top two sites, which was two to three times higher than the next most active site (65 [±13] bats/km^2^). At Charlie Heath Memorial Conservation Area, the two highest average site densities were six times higher than the next highest estimated site density in that area.

The highest estimated densities did not correlate with the maximum exit counts at the six conservation areas (Table 3). Although Indian Hills and Rebel's Cove Conservation Areas had the highest recorded exit counts (234 bats and 155 bats, respectively), they had some of the lowest average estimated densities using acoustics (56 [±8] bats/km^2^ and 52 [±6] bats/km^2^, respectively).

**TABLE 3:** Estimated Indiana bat density per km^2^ based on acoustic data collected over three summers (2019–2021), the average number of Indiana bat captures over two summers (2019 and 2021), and the maximum exit count at known Indiana bat roost trees over two summers (2019 and 2021) at six conservation areas in northeast Missouri.

Conservation area	Estimated density from acoustics (bats/km^2^)	Captures^a^	Maximum exit Count^a^
Atlanta‐Long Branch	64	6	48
Charlie Heath	60	6	14
Hidden Hollow	45	6	24
Indian Hills	56	16	234
Mineral Hills	85	7	97
Rebel's Cove	52	6	155

^a^ Captures and roost exit counts during a concurrent study at the same six conservation areas in northeast Missouri (Starbuck et al., 2020, 2022).

## 4 DISCUSSION

We estimated the density of Indiana bats across six conservation areas in northeastern Missouri using three years of passive acoustic survey data. Volancy period was the most plausible predictor of density, although the magnitude of change between periods varied by conservation area. Our site‐specific nightly density estimates were similar to known summer colony sizes of Indiana bats derived from exit counts at roosts in Missouri (Callahan et al., 1997) and to genetic mark–recapture estimates for a population of Indiana bats in central Indiana (Oyler‐McCance et al., 2018). These results show that the density equation is a viable index for assessing bat populations with passive acoustic data. However, several matters need to be considered for future bat density calculations.

It is encouraging that volancy period was such a strong predictor of Indiana bat population density because we expect a within‐year change in population size as juveniles become volant. In a two‐year acoustic survey of an Indiana bat maternity colony in central Indiana, Kaiser and O'Keefe (2015) found that detection probabilities were higher later in the summer and on warmer nights, presumably due to the influx of volant juveniles into the area sampled. Changes in detection rates from May to August are less likely to relate to changes in the adult population of Indiana bats, as spring migration for this species occurs in March and April, and fall migration occurs in September and October (Pettit & O'Keefe, 2017). Indiana bats give birth to one pup per year (Thomson, 1982). Juvenile mortality rates of similar bat species are estimated to range from 45% to 71% (Schorr & Siemers, 2021), so we expected the population to increase by more than 50% but less than 100% from pre‐ to post‐volancy. We saw a change in fitted density estimates ranging from −8% to 202% from pre‐ to post‐volancy across all six conservation areas (Figure 2). Hidden Hollow showed the lowest amount of change (−8%), which could mean that this is not an important roosting area and is used relatively consistently by foraging bats across the summer. In contrast, Indian Hills, which is larger, disconnected from other large forest patches, and contained numerous Indiana bat roosts (Starbuck et al., 2020, 2022) had the largest change from pre‐ to post‐volancy.

The range of density estimates varied greatly at each conservation area, which reflects variation in activity across nights and among sites. Differences in conservation area size, percent forest, and proximity to other forested areas could affect how Indiana bats use each area to forage and roost, which might explain the variation in the range of density estimates by conservation area. Sites within a conservation area varied in the amount of vegetative clutter, which could also partly explain the wide variation in density estimates by site. Indiana bat echolocation calls are more detectable in less cluttered environments, such as mature forests with high canopy closure and low amounts of understory vegetation (Kaiser & O'Keefe, 2015). Bats also alter their echolocation calls in response to vegetative clutter (Broders et al., 2004), which could affect the accuracy of bat call identification.

Many site‐specific density estimates exceeded our largest exit count of 234 bats, but the overall average density estimates for each conservation area were more plausible (Table 3). Misidentification of other species' echolocation calls as Indiana bats could inflate density estimates; however, other *Myotis* species are rarely captured in the conservation areas we surveyed (Starbuck et al., 2020, 2022) and our filter did not yield any false positives when applied to a test set that included calls of bats from other genera. Bat call counts are likely to be higher in narrower corridors where bats may pass by the detector multiple times, leading to inflated site‐specific estimates. Variability in site‐specific estimates of density could also relate to proximity of detectors to bat roosts and preferred foraging areas, none of which were known to us when we designed the study. Using a spatially balanced random sampling design for placing detectors, as described by Rodhouse et al. (2011), and later adopted by the NABat monitoring program (Loeb et al., 2015), could help to avoid such biases; however, decisions on detector placement will ultimately depend on availability of secure and clutter‐free survey points.

To increase confidence in density estimates made from acoustic data, we need more information on bat behavior and detector/microphone specifications to choose appropriate values for the density formula parameters (Marques, Thomas, et al., 2013). We know little about acoustic signal angles, that is, the angle of emission of the bat's echolocation call, although the generalized random encounter model is sensitive to this parameter (Milchram et al., 2019). A bat with a wider angle of emission would be easier to detect than a bat with a narrow range of emission, thus the density estimate is lessened for wider angles. For flight speed, we relied upon averaged estimates for Indiana bats and other bats similar in size while flying in various situations, including in the laboratory, or while migrating or foraging (see Table 1). We recommend combining thermal imagery and acoustic recordings to study how a wide array of bat species change their echolocation calls and flight speeds in response to varying amounts of clutter. The threshold and detection angle parameters in our density estimate equation depend on the make and model of the detector and microphone; a nuisance factor here is that the terms used by each company may vary. For example, for the EcoObs detector used by Milchram et al. (2019), “threshold” is an adjustable setting that refers to the distance at which the device is able to detect ultrasound; for the Anabat Swift, there is no threshold setting, so we estimated this value (−36 dB) based on the typical range at which these devices detect 40‐kHz ultrasound. More consistent nomenclature and settings would allow us to compare density estimates across devices. Our external microphone was directional, with a smaller perceived detection angle than an omnidirectional microphone. Directional microphones sacrifice detection angle for detection distance (Sprong et al., 2012), which is another factor we should consider in the detection distance equation.

Using passive acoustic data to estimate bat density would be a significant advancement for monitoring bat populations. Acoustic detectors can be used to survey a broader study area simultaneously and acoustic surveys require less labor and time compared to mist‐netting (see Introduction). Acoustic data have been applied in long‐term studies to assess bat population changes over time (e.g., Barlow et al., 2015; Browning et al., 2022; Rodhouse et al., 2019). The random‐encounter‐model approach (Milchram et al., 2019; this study) improves upon simply using raw acoustic data because it incorporates covariates related to bat flight and echolocation behavior, weather, and detector sensitivity. Considering these factors when estimating abundance will facilitate comparisons among bat species, different types of recording devices, and across different environmental conditions. This method may be best suited for studies where acoustic density estimates will illuminate population trajectories over long time periods (Marques et al., 2013). Future work could compare exit count data for known roosts with density estimates from nearby detectors. Concurrently deploying thermal or infrared cameras with acoustic detectors would give us information about detection rates and bat behaviors in the surveyed area (e.g., Gorresen et al., 2018). Comparing roost exit and camera data with acoustic detections will allow us to calibrate density estimates derived from acoustic surveys, thus increasing the efficacy of this approach for estimating bat populations.

## AUTHOR CONTRIBUTIONS

**Meredith L. Hoggatt:** Conceptualization (equal); data curation (equal); formal analysis (equal); investigation (lead); methodology (equal); project administration (equal); validation (equal); writing – original draft (lead); writing – review and editing (equal). **Clarissa A. Starbuck:** Data curation (equal); formal analysis (equal); investigation (equal); project administration (equal); validation (equal); visualization (equal); writing – review and editing (equal). **Joy M. O'Keefe:** Conceptualization (equal); funding acquisition (equal); investigation (equal); methodology (equal); project administration (equal); resources (equal); supervision (lead); validation (equal); writing – review and editing (equal).

## CONFLICT OF INTEREST STATEMENT

The authors have no conflicts of interest to declare.

## Data Availability

Data are available from the Illinois Data Bank: https://doi.org/10.13012/B2IDB‐7001459_V1.
